# RNA sequencing: new technologies and applications in cancer research

**DOI:** 10.1186/s13045-020-01005-x

**Published:** 2020-12-04

**Authors:** Mingye Hong, Shuang Tao, Ling Zhang, Li-Ting Diao, Xuanmei Huang, Shaohui Huang, Shu-Juan Xie, Zhen-Dong Xiao, Hua Zhang

**Affiliations:** 1 Institute of Laboratory Medicine, Guangdong Provincial Key Laboratory of Medical Molecular Diagnostics, School of Medical Technology, Guangdong Medical University, Dongguan, 523808 China; 2 Biotherapy Center, The Third Affiliated Hospital of Sun Yat-Sen University, Guangzhou, 510630 China; 3 Health Science Center, The University of Texas, Houston, 77030 USA

**Keywords:** RNA sequencing, Application, Cancer

## Abstract

Over the past few decades, RNA sequencing has significantly progressed, becoming a paramount approach for transcriptome profiling. The revolution from bulk RNA sequencing to single-molecular, single-cell and spatial transcriptome approaches has enabled increasingly accurate, individual cell resolution incorporated with spatial information. Cancer, a major malignant and heterogeneous lethal disease, remains an enormous challenge in medical research and clinical treatment. As a vital tool, RNA sequencing has been utilized in many aspects of cancer research and therapy, including biomarker discovery and characterization of cancer heterogeneity and evolution, drug resistance, cancer immune microenvironment and immunotherapy, cancer neoantigens and so on. In this review, the latest studies on RNA sequencing technology and their applications in cancer are summarized, and future challenges and opportunities for RNA sequencing technology in cancer applications are discussed.

## Background

Cancer remains one of the major malignant diseases that endangers human life and health and comprises complex biological systems that require accurate and comprehensive analysis. Since the first appearance of high-throughput sequencing in 2005 [1], it has become possible to understand life activities at the molecular level and to conduct detailed research to elucidate the genome and transcriptome. As an essential part of high-throughput sequencing, RNA sequencing (RNA-seq), especially single-cell RNA sequencing (scRNA-seq), provides biological information on a single tumor cell, analyzes the determinants of intratumor expression heterogeneity and identifies the molecular basis of formation of many oncological diseases [2, 3]. Thus, RNA sequencing offers invaluable insights for cancer research and treatment. With the advent of the era of precision medicine, RNA sequencing will be widely used for research on many different types of cancer. This review summarizes the history of the development of RNA sequencing and focuses on the latest studies of RNA sequencing technology in cancer applications, especially single-cell RNA sequencing and spatial transcriptome sequencing. In addition, we provide a general introduction to the current bioinformatics analysis tools used for RNA sequencing and discuss future challenges and opportunities for RNA sequencing technology in cancer applications.

## The development of RNA sequencing technologies

**Fig. 1:**
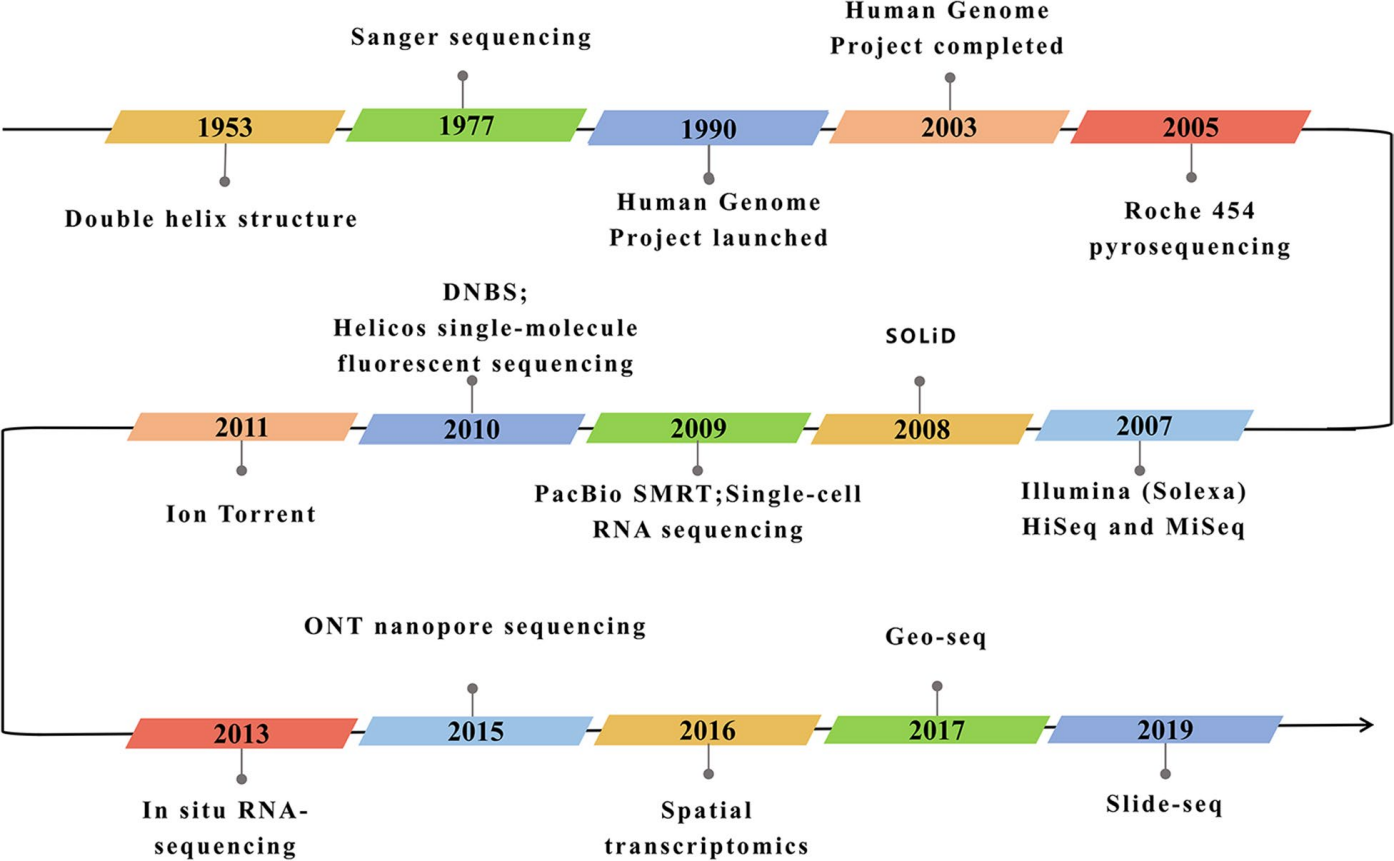
The development timeline of RNA sequencing technologies

It was not until 1953 when Watson and Crick proposed the double-helix structure did people truly realize at the molecular level that the essence of life is the result of gene interactions [4]. The continuous development of RNA sequencing has ushered transcriptome analysis into a new era, with higher efficiency and lower cost. The timeline of RNA sequencing technologies is shown in Fig. 1.

**Fig. 2:**
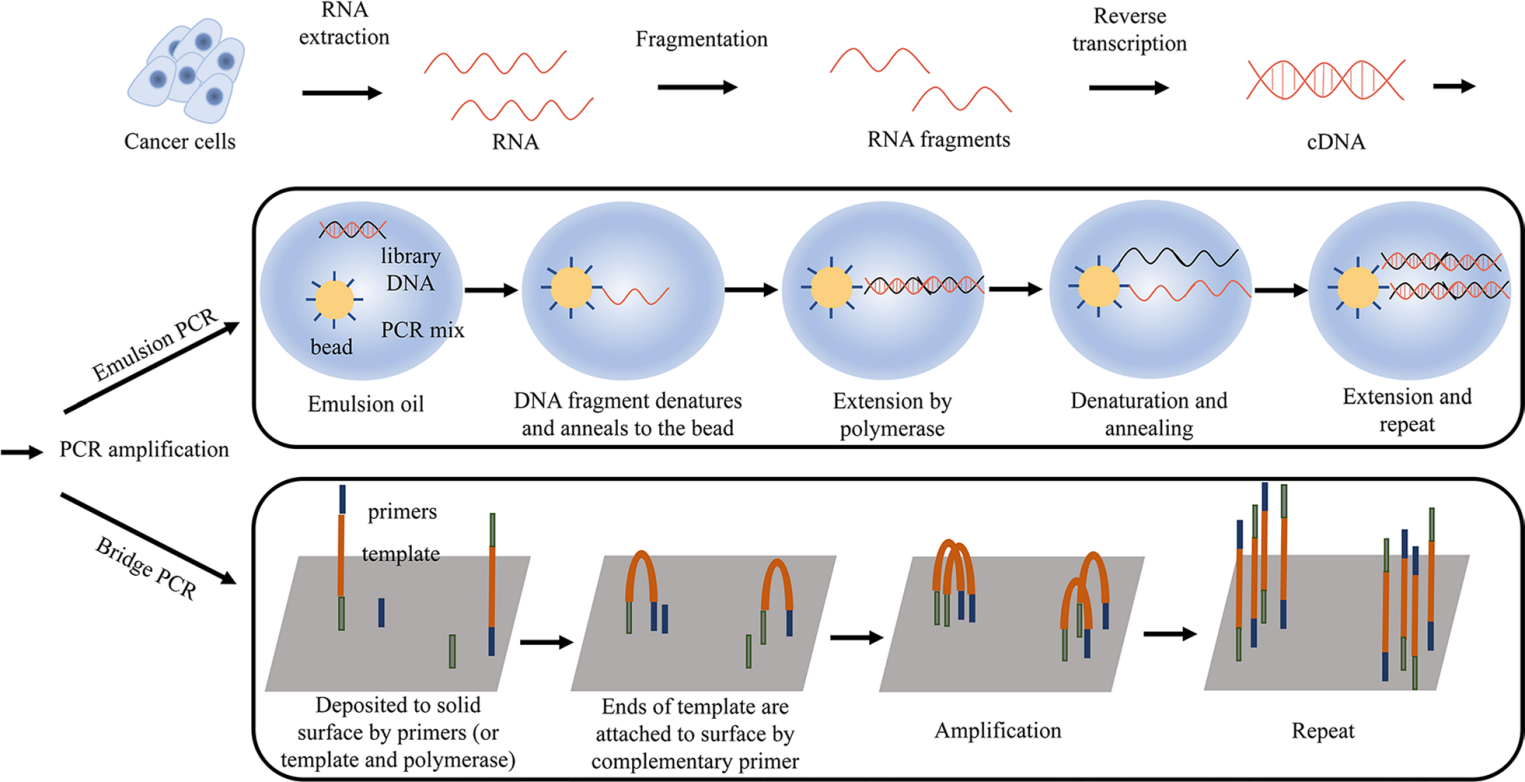
RNA extraction and template preparation before RNA-sequencing. RNA was extracted from tissues, and after fragmentation, fragmented DNA molecules were converted into cDNA by reverse transcription then amplified by emulsion PCR or bridge PCR to prepare sequencing library

**Fig. 3:**
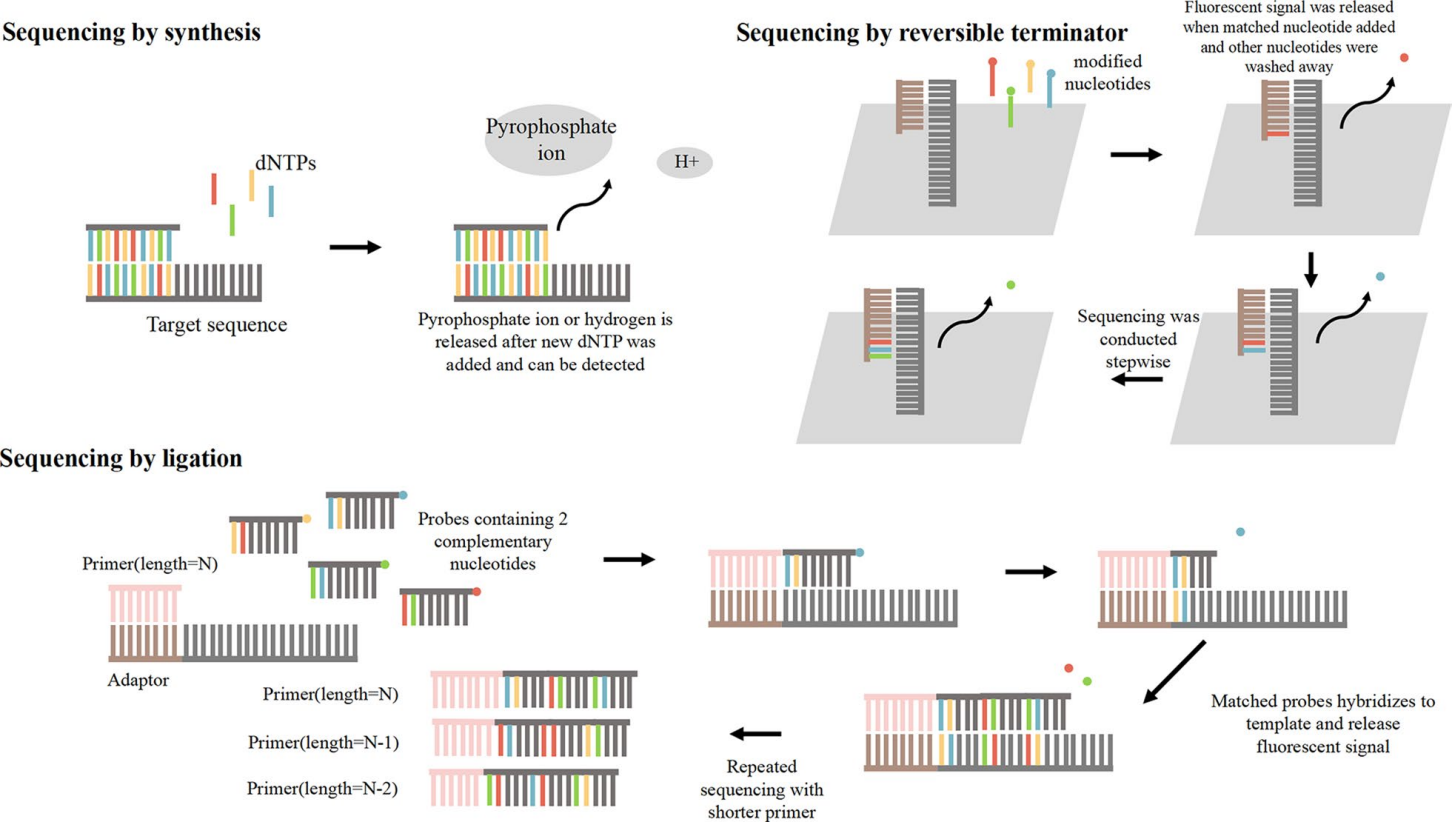
Three kinds of sequencing methods. These methods contain sequencing by synthesis, sequencing by reversible terminator and sequencing by ligation. And their different mechanisms are shown in detail

The first-generation sequencing technology is also called Sanger sequencing. The chain termination method was initiated by Sanger in 1977, followed by the chemical degradation method developed by Maxam and Gilbert [5, 6]. The same year, Sanger determined the 5368 bp genome of phage φX174, which is the first DNA genome sequenced [7]. The DNA microarray has aided significant progress in many fields since it was first introduced. However, microarrays require prior knowledge of gene sequences and are unable to identify novel gene expression [8]. After the first high-throughput sequencing platform appeared in 2005 [1], multiple next-generation sequencing platforms followed (Table 1, Figs. 2, 3). The accuracy and reproducibility among different platforms depended on several factors, including the inherent features of the platform and the corresponding analysis pipelines [9, 10]. Pyrosequencing that was no longer supported after 2016, developed by 454 Life Sciences, used a “sequencing by synthesis” method [1, 11–13]. The ion torrent sequencing platform is also based on the “sequencing by synthesis” method, which outperforms pyrosequencing with respect to sensitivity. SOLiD (Sequencing by Oligonucleotide Ligation and Detection) exhibits high accuracy, as each base is sequenced twice, but the read length is short [11–13]. DNBS (DNA nanoball sequencing) enables large collection of DNA nanoballs for simultaneous sequencing. Illumina-based sequencing technology represents a “reversible terminator sequencing” method. High-throughput sequencing has the advantage of fast speed, low sequencing cost and high accuracy, otherwise known as next-generation sequencing (NGS). Compared to microarray, it can detect unknown gene expression sequences but is time intensive [14].

**Table 1:** Comparison of different RNA sequencing platforms

Platform	Company	Read length(bases)	Run time	Volume per run	Cost	Template preparation	Sequencing chemistry
*The first-generation sequencing*							
Sanger	Life sciences	800 bp	2 h	1 read	$2400 per million bases	Bacterial cloning	Dideoxynucleosides terminator
*The next-generation sequencing*							
Roche 454 pyrosequencing	454 Life sciences	700 bp	< 24 h	0.7 Gb	$10 per million bases	Emulsion PCR	Sequencing by synthesis, pyrosequencing
Illumina HiSeq	Illumina	100 bp	3–10 days	120–1500 Gb	$0.02—$0.07 per million bases	Bridge PCR	Reversible terminator sequencing
Illumina MiSeq	Illumina	100 bp	1–2 days	0.3–15 Gb	$0.13 per million bases	Bridge PCR	Reversible terminator sequencing
SOLiD	Applied biosystems instruments (ABI)	50–75 bp	7–14 days	30 Gb	$0.13 per million bases	Emulsion PCR	Sequencing by ligation
DNA nanoball sequencing	Complete genomics	440–500 bp	9 days	20–60 Gb	$4400/genome	Rolling circle replication	Hybridization and ligation
Ion torrent	454 Life sciences	200–500 bp	4–5 h	660 Mb; 11 Mb	$300 to $750 per run	Emulsion PCR	Sequencing by synthesis
*The third-generation sequencing*							
SMRT	Pacific biosciences	> 900 bp	1-2 h	0.5–1 Gb	$2 per million bases	No need	Sequencing by synthesis
Helicos sequencing	Helicos biosciences	25–60 bp	8 days	21–35 Gb	$0.01 per million bases	No need	Hybridization and synthesis
Nanopore sequencing	Oxford nanopore technologies	Up to 98 kb	48/72 h	Up to 30 Gb	< $1 per million bases	No need	Nanopore

In addition to NGS, there is third-generation sequencing, which allows for long-read sequencing of individual RNA molecules [15]. Single-molecule RNA sequencing enables the generation of full-length cDNA transcripts without clonal amplification or transcript assembly. Thus, third-generation sequencing is free from the shortcomings generated by PCR amplification and read mapping. It can greatly reduce the false positive rate of splice sites and capture the diversity of transcript isoforms [15]. Single-molecule sequencing platforms comprise Pacific Biosciences (PacBio) single-molecule real-time (SMRT) sequencing [16], Helicos single-molecule fluorescent sequencing [17] and Oxford Nanopore Technologies (ONT) nanopore sequencing [18]. Furthermore, RNA-seq recently evolved from bulk sequencing to single-cell sequencing. Single-cell RNA sequencing was first published in 2009 to profile the transcriptome at single-cell resolution [19]. Drop-Seq and InDrop were initially reported in 2015 by analyzing mouse retina cell and embryonic stem cell transcriptomes, identifying novel cell types. Sci-RNA-seq, single-cell combinatorial indexing RNA sequencing, was developed in 2017, and SPLiT-seq (split-pool ligation-based transcriptome sequencing) was first reported in 2018. Both approaches use a combinatorial indexing strategy in which attached RNAs are labeled with barcodes that indicate their cellular origin [20, 21].

Though single-cell data enable single-cell transcriptomics, it may lose spatial information during single-cell isolation. To solve this problem, spatial transcriptomics has emerged. Spatial transcriptomics employs unique positional barcodes to visualize RNA distributions in RNA sequencing of tissue sections and was first published in 2016 [22]. Slide-seq, reported in 2019, uses DNA barcode beads with specific positional information [23]. Geo-seq was introduced in 2017 and integrated scRNA-seq with laser capture microdissection (LCM), which can isolate individual cells [24]. In situ sequencing refers to targeted sequencing of RNA fragments in morphologically preserved tissues or cells without RNA extraction, including in situ cDNA synthesis by padlock probes or stably cross-linked cDNA amplicons in fluorescent in situ RNA sequencing (FISSEQ) and in situ amplification by rolling-circle amplification (RCA) [25, 26]. Furthermore, various new technologies based on RNA-seq have been developed for specific applications. For example, a type of targeted RNA sequencing, CaptureSeq, employs biotinylated oligonucleotide probes and results in the enrichment of certain transcripts to identify gene fusion [27, 28].

## Computational analysis of RNA sequencing data

**Fig. 4:**
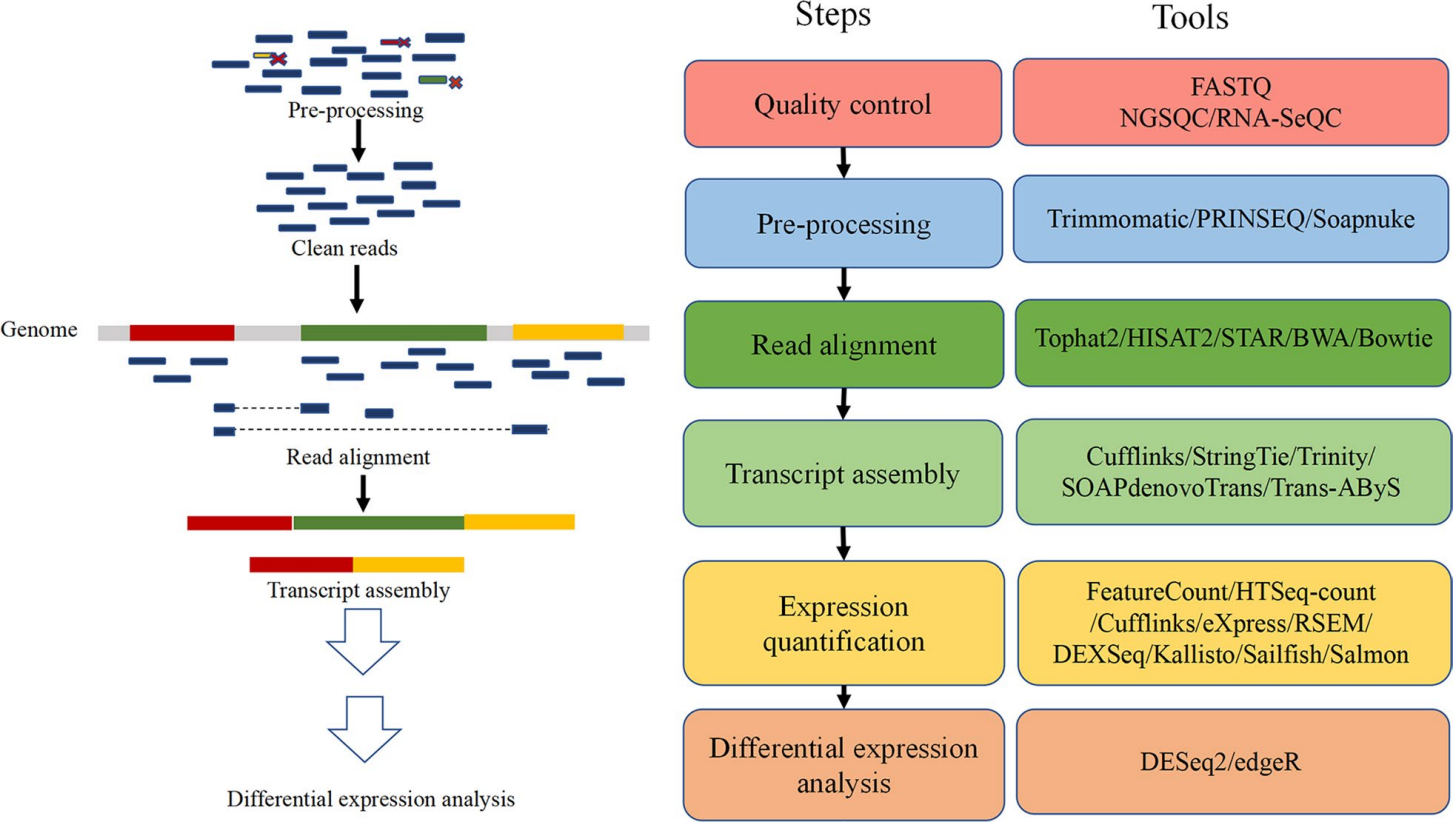
Bioinformatics tools commonly used in RNA-seq data analysis. These tools are primarily used in the four main processes of RNA-seq data analysis, including quality control, read alignment and transcript assembly, expression quantification and differential expression analysis

Computational analysis tools for RNA sequencing have dramatically increased during the past decade. The choice of a particular tool should be based on the purpose and accuracy of application [29–31]. A general RNA sequencing data analysis process involves the quality control of raw data, read alignment and transcript assembly, expression quantification and differential expression analysis (Fig. 4).

The first step of data analysis is to assess and clean the raw sequencing data, which is usually provided in the form of FASTQ files [32]. Quality control visually reflects the quality of the sequencing and purposefully discards low-quality reads, eliminates poor-quality bases and trims adaptor sequences [31]. Common tools include FASTQ [33], NGSQC [34], RNA-SeQC [35], Trimmomatic [36], PRINSEQ [37] and Soapnuke [38].

The next step is to map the clean reads to either a genome or a transcriptome. There are some mapping tools available, including Tophat2 [39], HISAT2 [40], STAR [41], BWA [42] and Bowtie [43]. After alignment, another type of software, such as Cufflinks [44], StringTie [45], Trinity [46], SOAPdenovoTrans [47] and Trans-AByS [48] can be used to assemble transcripts from short-reads. When the transcript model is established, its expression can be quantified at the gene, transcript and exon levels. Commonly used software for gene-level quantification includes FeatureCount [49] and HTSeq-count [50]. Transcript level quantitative software includes Cufflinks [44], eXpress [51] and RSEM [52]. DEXSeq is a software for exon level quantification [53]. In addition, there are some alignment-free quantification tools such as Kallisto [54], Sailfish [55] and Salmon [56], which have the advantage of marked computational resource saving. After normalizing, an expression matrix is generated, and statistical methods can be used to identify differentially expressed genes. DESeq2 [57] and edgeR [58] are commonly used to perform this task.

### Applications of RNA-sequencing in cancer research

**Fig. 5:**
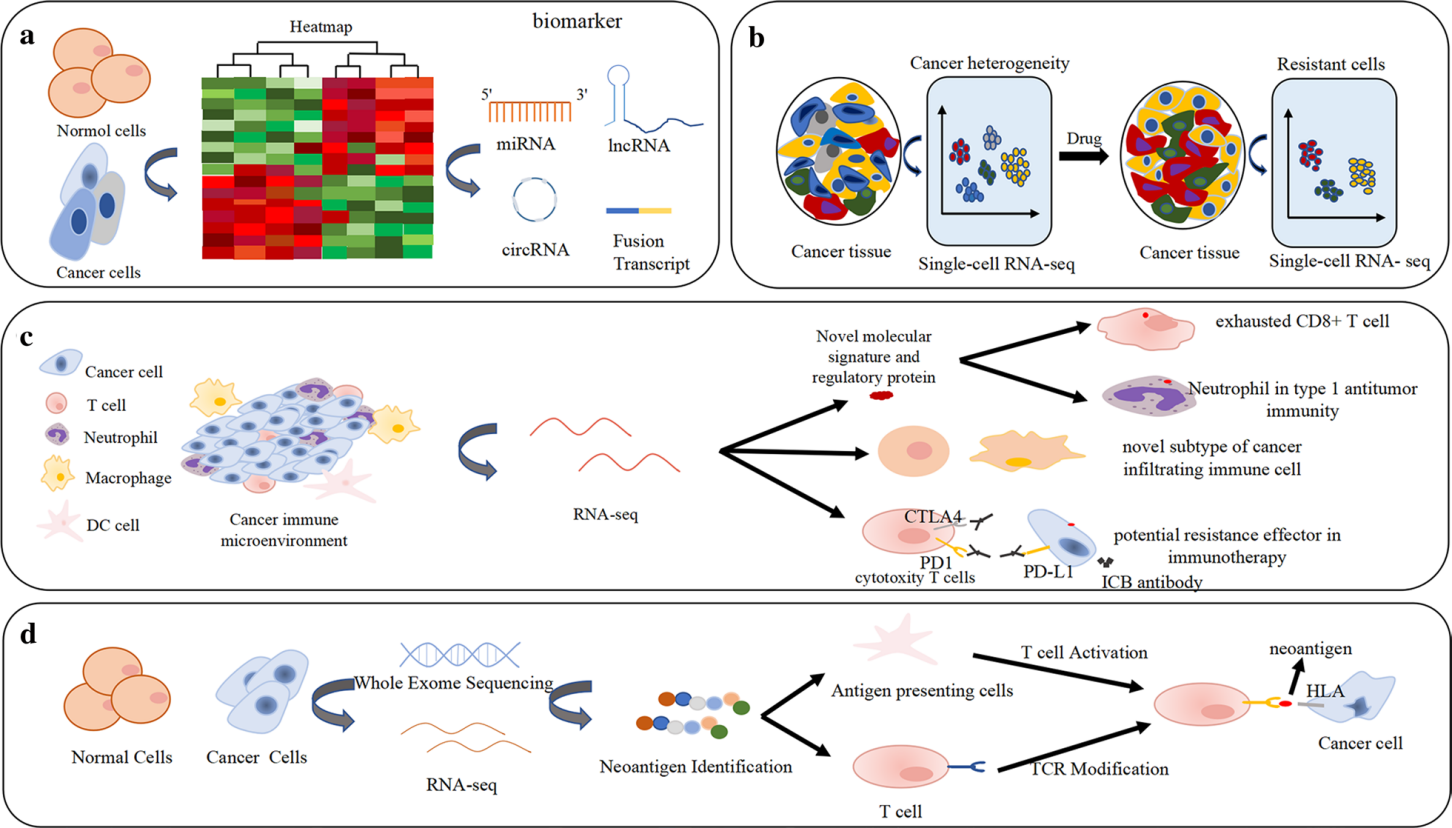
Applications of RNA-seq in differential expression analysis and cancer biomarkers, cancer heterogeneity and drug resistance, cancer immune microenvironment, immunotherapy and neoantigen. **a** Differential expression analysis by RNA sequencing can identify potential biomarkers, including fusion transcript, lncRNA, miRNA and circRNA. **b** The heterogeneity and drug resistance of cancer cells identified by RNA-seq. **c** Novel molecular signature, regulatory protein and unknown subtypes in cancer infiltrating immune cells and potential resistance effector in immunotherapy can be identified by RNA-seq; **d** Neoantigen profiling by RNA-seq and TCR modification targeted neoantigens

Genomic data, such as RNA-seq, have become widely available due to the popularity of high-throughput sequencing technology [59]. As an important part of next-generation sequencing, RNA sequencing has made great contributions in various fields, especially cancer research, including studies on differential gene expression analysis and cancer biomarkers, cancer heterogeneity and evolution, cancer drug resistance, the cancer microenvironment and immunotherapy, neoantigens, etc. (Fig. 5).

### Differential gene expression analysis and cancer biomarkers

Differential gene expression analysis is one of the most common applications of RNA sequencing [60]. Samples from different backgrounds (different species, tissues and periods) can be used for RNA sequencing to identify differentially expressed genes, revealing their function and potential molecular mechanisms [61]. More importantly, differential gene expression analysis facilitates the discovery of potential cancer biomarkers [62]. Many studies have shown that gene fusions are closely related to oncogenesis and are appreciated as both ideal cancer biomarkers and therapeutic targets [63]. Gene fusions in clinical samples are primarily detected by RNA-CaptureSeq. Compared to whole transcriptome sequencing, RNA-CaptureSeq has significantly higher sequencing depth [27, 64, 65]. It has been reported that the NUP98-PHF23 fusion gene is likely to be a novel therapeutic target in acute myeloid leukemia (AML) [66]. Recently, a variety of recurrent gene fusions, including ESR1-CCDC170, SEC16A-NOTCH1, SEC22B-NOTCH2 and ESR1-YAP1, have been identified in breast cancer, indicating that recurrent gene fusion is one of the key drivers for cancer [67]. Several novel configurations of  BRAF, NTRK3 and RET gene fusions have been identified in colorectal cancer [68]. These fusions may promote the development of malignancy and provide new targets for personalized treatment [68]. In addition, some special genomic factors have been discovered as biomarkers by RNA sequencing, including miRNA, lncRNA and circRNA, which are widely present in various types of cancer [69–71]. A recent example is circRNA_0001178 and circRNA_0000826, which are biomarkers of colorectal cancer metastasis to the liver [72]. By applying both RNA sequencing and small RNA sequencing, a study on pancreatic cancer identified differential expression of simple repetitive sequences (SSRs) and demonstrated that the frequency of SSR motifs changed dramatically, which is expected to become a tumor biomarker [73]. In addition to nucleic acid biomarkers, RNA-seq combined with immunohistochemistry and western blot has also identified certain proteins as cancer biomarkers, such as nuclear COX2 (cyclooxygenase2) in combination with HER2 (human epidermal growth factor receptor type 2), which may serve as potential biomarkers for the diagnosis and prognosis of colorectal cancer [74]. Similar examples identified using RNA-seq profiling analysis include ISG15 (Interferon-stimulated gene 15) in nasopharyngeal carcinoma [75] and DMGDH (dimethylglycine dehydrogenase) in hepatocellular carcinoma [76]. Data-mining analysis of RNA sequencing data and other clinical data has identified that the isoforms of peroxiredoxins also can be expected the prognostic biomarkers for predicting overall survival and relapse-free survival in breast cancer [77]. Increasing differentially expressed genes are being identified by RNA sequencing, and new potential cancer biomarkers are being continuously discovered (Table 2). However, sufficient clinical practice is needed to confirm the diagnostic and predictive applications of these biomarkers in cancer.

**Table 2:** Representative potential biomarkers identified by RNA-seq in cancer

Cancer type	Biomarker name	Biomarker type	Up/Down	Value	References
Liver cancer	tRNA-ValTAC-3/tRNA-GlyTCC-5/tRNA-ValAAC-5/tRNA-GluCTC-5	tsRNA	Up	Diagnostic	[92]
	ACVR2B-AS1	LncRNA	Up	Prognostic/therapeutic target	[93]
Lung cancer	LINC01537	LncRNA	Down	Prognostic/therapeutic target	[94]
	circFARSA	CircRNA	Up	Noninvasive biomarker	[95]
	LINC01123	LncRNA	Up	Prognostic/therapeutic target	[96]
Gastric cancer	CTD2510F5.4	LncRNA	Up	Diagnostic/prognostic	[97]
	MEF2C-AS1/FENDRR	LncRNA	Down	Diagnostic/prognostic	[98]
Prostate cancer	PSLNR	LncRNA	Down	Diagnostic/therapeutic target	[99]
Colorectal cancer	RAMS11	LncRNA	Up	Therapeutic target	[100]
	CRCAL-1/CRCAL-2 /CRCAL-3/ CRCAL-4	LncRNA	Up	Therapeutic target	[101]
Colon cancer	AFAP1-AS1	LncRNA	Up	Prognostic/ therapeutic target	[102]
Head and neck squamous cell carcinoma	LINC00460	LncRNA	Up	Prognostic	[103]
	HCG22	LncRNA	Down	Prognostic	[104]
	HOXA11-AS/LINC00964/MALAT1	LncRNA	Up	Diagnostic	[105]
Clear cell renal cell carcinomas	SLINKY	LncRNA	Up	Prognostic	[106]
Leukemia	LUCAT1	LncRNA	Up	Therapeutic target	[107]
	circ-HIPK2	CircRNA	Down	Diagnostic/prognostic	[108]

RNA-seq could detect early mutations as well as high molecular risk mutations, thus can discover novel cancer biomarkers and potential therapeutic targets, monitoring of diseases and guiding targeted therapy during early treatment decisions. Tumor mutation burden (TMB) is considered as a potential biomarker for immune checkpoint therapy and prognosis [78, 79]. RNA-seq can be used to explore the application value of TMB in diffuse glioma [78]. Through the RNA-seq, MET exon 14 mutation and isocitrate dehydrogenase 1 (IDH1) mutation were identified as new potential therapeutic targets in lung adenocarcinoma and chondrosarcoma patients, respectively [80, 81]. Several studies have shown that RNA sequencing can effectively improve the detection rate on the basis of DNA sequencing, provide more comprehensive detection results and achieve a better curative effect for targeted therapy [82]. In addition, it has been proved that IDH mutation is a good prognostic marker for glioma by RNA-seq [83]. Targeted therapy is also considered to enhance or replace cytotoxic chemotherapy regimen in cancer including AML [84–86].

ScRNA-seq also has some new discoveries in diagnosis. For example, scRNA-seq data can be used to infer copy number variations (CNV) and to distinguish malignant from non-malignant cells. The infer CNV algorithm, which was used in the study of glioblastoma, uses averaging relative expression levels over large genomic regions to infer chromosome copy number variation [87]. Similar examples include head and neck cancer [88] and human oligodendroglioma [89]. It is reported that RNA sequence of tumor-educated blood platelets (TEPs) can also become a blood-based cancer diagnosis method [90]. It should be noted that the lack of detailed functional implications of the identified RNAs in platelets in the field of platelet RNA research is also an urgent problem to be solved [91].

### Cancer heterogeneity and evolution

Heterogeneity has always existed during the transformation of normal cells to cancer cells. The continuous accumulation of heterogeneity may reflect the evolution of cancer [109]. Early RNA sequencing detected all RNA transcripts in a given tissue or cell group, ignoring differences in individual cells. Transcriptome profiling of single-cell RNA sequencing solves this problem by providing single-cell resolution of the transcriptome [3]. In melanoma, single-cell RNA-seq was used to analyze 4645 tumor cells from 19 patients, including cancer cells, immune cells, mesenchymal cells and endothelial cells. Transcriptomic data from different single cells revealed that heterogeneity of cells within the same cancer is associated with cell cycle, spatial background and drug resistance [110]. A recent single-cell RNA-seq study of 49 samples of metastatic lung cancer revealed changes in plasticity induced by non-small cell lung cancer treatment, providing new directions for clinical treatment [111]. Single-cell RNA sequencing also integrates a variety of information in a single cancer cell, deciphering the secrets of cancer heterogeneity and evolution [112]. Compared with scRNA-seq, another emerging technology spatial transcriptome sequencing incorporates information on the spatial location of cells. In prostate cancer, using spatial transcriptomics technology, the transcriptome of nearly 6750 tissue regions was analyzed, revealing the whole-tissue gene expression heterogeneity of the entire multifocal prostate cancer and accurately describing the range of cancer foci [113]. In a study of breast cancer tissues, the results of spatial transcriptome sequencing revealed that gene expression among different regions was surprisingly highly heterogeneous [22]. In recent years, single-nucleus RNA sequencing (snRNA-seq) has also received extensive attention due to its solving the problem that single-cell RNA sequencing cannot be applied to frozen specimens and cannot obtain all cell types in a given tissue [114, 115]. The emerging technology of RNA sequencing will contribute to research on cancer heterogeneity and evolution.

### Cancer drug resistance

Drug resistance is a main reason leading to cancer treatment failure. However, the molecular mechanisms underlying drug resistance are still poorly understood [116]. RNA sequencing became a vital tool for revealing the mechanisms of cancer drug resistance. In breast cancer, single-cell RNA sequencing identified a tumor-infiltrating immunosuppressive immature myeloid cell that leads to drug resistance [117]. Another study identified a new COX7B gene related to platinum resistance and a surrogate marker CD63 in cancer cells by single-cell RNA-seq [118]. RNA sequencing has also demonstrated that cancer cells that wake up from a dormant state produce large amounts of BORIS (brother of the regulator of imprinted sites), which can regulate the expression of survival genes in drug-resistant neuroblastoma cells [119]. Identifying special molecules that mediate these processes could help us understand the occurrence of drug resistance. Single-cell transcriptomics can be used to study different modes of chemoresistance in tumor cells and has shown that pre-existing drug-resistant cells can be selected through higher phenotypic intratumoral heterogeneity, while phenotypic homogeneous cells use other mechanisms to trans-differentiate under drug-selection [120]. In one study of pancreatic ductal adenocarcinoma, human pancreatic cancer (PANC-1) cells and gemcitabine-resistant PANC-1 cell lines were compared by RNA sequencing, and two circRNAs were identified as both novel biomarkers and potential therapeutic targets for gemcitabine resistant patients [121]. RNA-seq has also conducted in-depth research on the drug resistance of hematological malignancies. Through RNA-seq, it has been found that non-coding RNAs and fusion genes play an important role in mediating the drug resistance of hematological malignancies [122]. A good example is to compare the circRNA expression profile of the drug-resistant acute myeloid leukemia cell with its parent cell, and determine the circRNAs involved in drug resistance [123]. Similarly, the novel MEF2D-BCL9 fusion transcript identified by RNA-seq was found to increase HDAC9 (histone deacetylase 9) expression and to enhance the resistance to dexamethasone in acute lymphocytic leukemia (ALL) [124]. Leukemia stem cells (LSCs), a rare cell population assumed to be responsible for relapse, is crucial to improve the prognosis of patients [125, 126]. RNA-seq analysis showed that LSCs have a unique lncRNA signature with functional relevance and therapeutic potential, providing an explanation for chemotherapy resistance and disease recurrence [127].

### The cancer microenvironment and immunotherapy

The immune system plays a critical role in the cancer microenvironment, affecting several stages of cancer development, including tumorigenesis, progression and metastasis, through tumor-infiltrating lymphocytes (TILs) [128]. TILs and their interactions with malignant cells and stromal cells make up the cancer immune microenvironment. Due to the heterogeneity of cancer, it is difficult to define the exact pro- or anti-cancer function of certain immune cells. Cancer heterogeneity also causes the varied clinical efficacy observed in patients treated with immunotherapies due to different responses of different subclones [129]. Transcriptomic profiling by RNA-seq, in particular scRNA-seq, provides comprehensive information on cellular activity and interactions among cells in the tumor microenvironment (TME). ScRNA-seq enables genomic and molecular profiling of high quantity and quality individual immune cells and assessment of cellular heterogeneity to depict the immune system spectrum in the cancer microenvironment [130–132]. ScRNA-seq data demonstrated that compared to normal tissues, cancer tissues exhibited significantly higher heterogeneity in the immune microenvironment, and a continuity in T cell activation resulting from polyclonal T cells and heterogeneous antigen-presenting cells has been identified [133].

ScRNA-seq of tumor-infiltrating T cells in metastatic melanoma identified transcription factor NFATC1 (nuclear factor of activated T cells 1) as a potential molecular signature of T cell exhaustion programs and revealed the depletion of low-exhaustion T cells in expanded clones of T cells [110]. Combining scRNA-seq with assembled T cell receptor (TCR) sequences, 11 T cell subsets, such as CD8 ^+^ T cells and CD8^ +^ FOXP3 ^+^ regulatory-like cells, and their genomic signatures, were identified in hepatocellular carcinoma (HCC), providing valuable insights for understanding the immune landscape of infiltrating T cells in HCC [134]. Cancer infiltrating T cells also play an anti-tumor role through impairment of an autophagy protein, LC3 (microtubule-associated protein 1A/1B-light chain 3, often short for LC3)-associated phagocytosis (LAP), demonstrating the role of autophagy in oncogenesis and suppression revealed by scRNA-seq [135]. In addition to solid tumors, scRNA-seq of acute myeloid leukemia patients detected diverse immunomodulatory genes that suppress T cell function [136]. By CSOmap, a computational tool for scRNA-seq, the CCL4-CCR8 directed interaction between Tregs and Texs, as well as reduced proliferation of Texs, was characterized [137]. Notably, findings also revealed that tumor-infiltrating T cells exhibited more interactions among themselves than with T cells from peripheral blood and different interactions between tumors and T cells, indicating a varied response to immunotherapy and a potential trend for immune escape [137].

In the cancer immune microenvironment, neutrophils, in addition to T cells, are also key components of cancer progression and cancer drug resistance [138–141]. Through scRNA-seq of murine sarcomas and certain human cancers, neutrophils with CSF3R (colony stimulating factor 3 receptor) expression were found to be a part of type 1 antitumor immunity associated with unconventional CD4^−^CD8^−^αβ T cells (UTCαβ) in anti-cancer immunity, indicating better prognosis [142]. ScRNA-seq of metastatic breast cancer and CD45 cells from primary cancer identified neutrophils as pro- and anti-tumorigenic or metastatic, in which pro-tumorigenic and metastatic neutrophils are induced by IL11 expressing cancer subclones, resulting in polyclonal metastasis [143]. This observation also provides new insight into anti-cancer immunotherapy by targeting neutrophils [143]. With scRNA-seq of CD4 and CD8 T cells, several crucial pathways with anti-cancer function were revealed [144].

The balance between immune reaction and immune tolerance is the basis of immune homeostasis, which is also involved in anti-cancer immunity and oncogenesis. scRNA-seq of monocytes and dendritic cells (DCs) separated from a single lymph node melanoma metastasis revealed a conserved homeostatic module regulated by suppressor-of-cytokine-2 (SOCS2) protein and IFNγ [145]. SOCS2 serves an essential regulatory role in anti-tumor immunity and T cell priming through DCs. This highly conserved homeostatic program establishes a connection between autoimmune prevention and immune surveillance in cancer [145].

Immunotherapies, especially immune checkpoint blockade (ICB), has opened a new chapter for anti-cancer therapy with remarkable responses from targeting programmed death 1 (PD1), programmed death-ligand 1 (PD-L1) and cytotoxic T-lymphocyte-associated protein 4 (CTLA4) [146–148]. However, only a few patients benefit from ICB, and severe side effects were observed [149, 150]. Obviously, various unknown determinants are correlated with the outcome of immunotherapies in addition to well-known factors such as PD1/PD-L1/CTLA-4 expression and mismatch repair deficiency [151–155]. Therefore, it is paramount to identify potential effectors for ICB efficacy. By analyzing RNA-seq data from melanoma patients who underwent anti-PD1 and anti-CTLA4 treatment, a potential ICB resistance effector SERPINB9 (a member of the serine protease inhibitor (serpin) family) and the connection between cytotoxic T lymphocytes (CTL) infiltration level and ICB response were characterized [156]. A sub-population cells with immunotherapy persistence have been identified by scRNA-seq and were found to have stem cell-like states with the expression of stem cell antigen-1 (Sca-1) and Snai1 [157].

Another immunotherapy, myeloid-targeted immunotherapy, is based on the complexity of tumor-infiltrating myeloid cells, including DCs and tumor-associated macrophages (TAMs) revealed by scRNA-seq [158]. Through scRNA-seq of immune cells from colorectal cancer patients, C1QC^ +^ and SPP1^ +^ TAMs, two subsets of TAMs, were identified, and the mechanism of myeloid-targeted immunotherapy, such as anti-CSF1R (colony stimulating factor 1 receptor) and CD40 agonist, was revealed [159]. Intracellular staining and sequencing (INs-seq), a novel technology integrating scRNA-seq and intracellular protein activity measurements, revealed novel Arg1^ +^ Trem2 ^+^ regulatory myeloid (Mreg) cells and demonstrated that depletion of Trem2 led to deduction of exhausted CD8 T cells with increased NK and cytotoxic T cells and cancer suppression by reducing accumulation of intratumoral Mreg cells [160].

### Cancer neoantigens

Neoantigens, human leukocyte antigen (HLA)-bound peptides derived from cancer-specific somatic mutations or gene fusions during tumor growth, are another crucial regulator of the clinical response to immunotherapy [161]. Higher intratumor neoantigen heterogeneity and clonal neoantigen burden increases sensitivity to ICB and contributes to better clinical outcome in patients with melanoma and advanced non-small cell lung cancer [162]. This kind of antigen is an optimal target for anti-cancer immunotherapy, enhancing neoantigen-specific T cell activity, and a vaccine targeting personal neoantigens for melanoma patients has been developed [163, 164]. Given all these promising features of personalized medicine targeting neoantigens in tumors, massive parallel profiling of tumor neoantigen burden is necessary for improving clinical efficacy and a deeper understanding of the neoantigen landscape. An RNA-seq-based transcriptomic approach is an efficient tool for neoantigen profiling in many studies. It was revealed that homology of neoantigen and somatic-mutation induced pathogens are important in response prediction in anti-CTLA4 treated melanoma [165]. In addition to melanoma, other studies found reduced neoantigen load in triple-negative and HER2 breast cancers [166], diverse neoantigen abundance in non-small-cell lung cancer patients with different treatment strategies [167], a decreased ratio of neoantigen expression to predicted neoantigens in recurrent glioma due to immune selection pressure [168], a negative correlation between neoantigen abundance and clinical outcome in selected solid tumors [169] and different neoantigen landscapes in immune filtration and T cell dysfunction based on histology in salivary gland carcinoma (SGC) patients [170].

A neoantigen prediction program, Neopepsee, based on RNA-seq data and somatic mutation, can be utilized to detect potential neoantigens for personal vaccine development with reduced false-positive rate compared to binding affinity prediction [171]. ScanNeo is another prediction computational pipeline based on RNA-seq that aims to identify insertion and deletion derived neoantigens, which was validated in prostate cancer [172], and ASNEO, which identifies personal-specific alternative splicing derived neoantigens [173]. Several neoantigens have been identified to be related to cancer prognosis and might be potential targets of immunotherapies, such as the TP53 neoantigen for HCC patients [174]. For the anti-neoantigen immunotherapy in cancer, a new strategy involving neoantigen-specific TCRs modification has been proposed, and scRNA-seq has been applied to isolate neoantigen-specific TCRs for further clinical application [175].

## Conclusions and perspectives

High-throughput RNA-seq technology has been a major tool to explore the transcriptome. The rapid development of RNA-seq technology not only saves time and cost but also sheds light on many new research fields. However, there are still limitations of RNA-seq technology that need to be improved.

For short-read length RNA-seq technologies, bias and imperfections are primarily generated in sequencing library preparation and short read assembly. It is difficult for these methods to correctly identify multiple isoforms from a certain gene. To overcome the disadvantage of short read length, improved read coverage and sequencing depth is required. Long-read length RNA-seq technologies avoid shortcomings in template amplification, reduce the false positive rate in splice junction detection and enable the identification of unannotated longer transcripts, overcoming the common limitations of short-read sequencing [176, 177]. However, this method suffers from the drawback of reduced throughput, higher cost and higher sequencing error rate, especially insertion-deletion errors. To reduce random errors, PacBio circular consensus-sequencing (CCS) was developed to increase sequencing depth by rereading molecules several times. However, it also reduces the identification rate of unique isoforms. In addition, the sensitivity of long-read sequencing for identification of differentially expressed genes is lower compared to short-read sequencing [178–180]. Thus, hybridization of long-read and short-read sequencing has been reported to yield a more comprehensive and accurate analysis [181].

Improvements in the throughput of RNA sequencing technology have resulted in billions of sequencing reads, bringing great challenges to the computational process, such as data storage, transmission, quality control and data analysis, including read mapping, transcript assembly and read normalization. Therefore, it is important for bioinformatics to keep pace with the continuous developments of RNA-seq technologies. Notably, bias could be produced due to differences in read data handling, necessitating the improvement of current bioinformatics pipelines.

RNA-seq measures gene expression by the read counts, which always containing missing values, thus results in information loss of specific gene and negative impact on downstream analysis. To overcome this problem, missing data need to be imputed and analyzed by several methods, such as optimal clustering with missing values [182]. For scRNA-seq, the proportion of genes with zero or low expression varies across cells due to biological or technical bias. For example, batch effects can come from cells captured and sequenced in different conditions [183]. Imputation methods, such as SAVER, MAGIC and kNN-smoothing, are recommended for scRNA-seq [184]. Another method named batch effects correction with unknown subtypes for scRNA-seq data (BUSseq) utilizes Bayesian hierarchical model and can also be used to correct batch effects and missing data [185].

Combination of data from multi-omics sequencing can undoubtedly expand the application of RNA-seq. For example, Assay for Transposase-Accessible Chromatin using sequencing (ATAC-seq) was developed by utilizing hyperactive Tn5 transposase to identify open chromatin region and transcriptional factor (TF) binding sites [186]. The integration of ATAC-seq and RNA-seq enables the reveal of TF-targeted genes and their transcripts [187, 188]. Chromatin conformation capture analysis (3C) technology and its several derivatives including circular chromosome conformation capture (4C), carbon copy chromosome conformation capture (5C), ChIP-Loop, Hi-C and capture Hi-C were developed and improved to detect chromatin structure as well as unknown interacting regions [189–191]. It has been reported that combined analysis of RNA-seq and chromatin structure can detect structure variation-related differentially expressed genes [192–194].

Epitranscriptomics is a crucial part of gene expression, and methylation of adenosine at the N6 position (m6A) is the most abundant [195]. Traditional RNA-seq needs reverse transcription before sequencing and thus easily loses the information of transcriptome complexity. This shortcoming can be overcome by directly sequencing native RNA molecules using methods such as nanopore sequencing. Transcript modifications could be inferred from the current signal as the modified RNA molecules passing nanopore cause a characteristic temporary current blockade, which enables the detection of diverse modifications such as m6A or 5-methylcytosine (m5C) [196–198].

ScRNA-seq is a powerful technology to facilitate further exploration in cancer research and also has been employed in the detection of cancer stem cell subpopulation, metabolic switch in cancer-draining lymph nodes and therapy-induced adaption of cancer cells [111, 199, 200]. Combined with cell sorting or ligand-receptor interaction, scRNA-seq was utilized in cellular interaction, cell spatial organization as well as molecular crosstalk characterization [137, 201, 202]. Coupling of parallel CRISPR (clustered regularly interspaced short palindromic repeats)-pooled screen, scRNA-seq enables the simultaneous analysis of genomic perturbation and transcriptional activity to detect heterogeneous cell type as well as crucial factors of complexity regulatory mechanism [203–205]. ScNT-seq, single-cell metabolically labeled new RNA tagging sequencing, brings RNA-seq into time resolution by identifying RNAs transcribed at different stage [206]. Utilizing SNP-based demultiplexing of scRNA-seq data, MIX-Seq was developed to study cancer cell reaction to pharmacologic treatment [207]. Another technology, snRNA-seq, is invaluable for detecting cellular heterogeneity of cancer and has been employed to identification of a sub-population of adipocytes regulating cancer genesis [208].

Taken together, RNA-seq has been applied in an impressively wide range of cancer research. All applications in cancer research rely on the boost of advanced RNA-seq technologies, especially the combination of scRNA-seq and spatial transcriptomics as well as data from multi-omics, which will bring RNA-seq technologies into single-cell resolution and tissue-level transcriptomics, providing new insight into cancer diagnosis, treatment and prevention.

## Data Availability

Not applicable.

## Abbreviations

RNA-seq: RNA sequencing
ScRNA-seq: Single-cell RNA sequencing
SOLiD: Sequencing by Oligonucleotide Ligation and Detection
DNBS: DNA nanoball sequencing
NGS: Next-generation sequencing
PacBio SMRT: Pacific Biosciences single-molecule real-time
ONT: Oxford Nanopore Technologies
Sci-RNA-seq: Single-cell combinatorial indexing RNA sequencing
SPLiT-seq: Split-pool ligation-based transcriptome sequencing
LCM: Laser capture microdissection
FISSEQ: Fluorescent in situ RNA sequencing
RCA: Rolling-circle amplification
AML: Acute myeloid leukemia
SSRs: Simple repetitive sequences
COX2: Cyclooxygenase2
HER2: Human epidermal growth factor receptor type 2
ISG15: Interferon-stimulated gene 15
DMGDH: Dimethylglycine dehydrogenase
TMB: Tumor mutation burden
IDH: Isocitrate dehydrogenase
CNV: Copy number variations
TEPs: Tumor-educated blood platelets
SnRNA-seq: Single-nucleus RNA-sequencing
BORIS: Brother of the regulator of imprinted sites
ALL: Acute lymphocytic leukemia
LSCs: Leukemia stem cells
TILs: Tumor-infiltrating lymphocytes
TME: Tumor microenvironment
NFATC1: Nuclear factor of activated T cells 1
TCR: T cell receptor
HCC: Hepatocellular carcinoma
LAP: Microtubule-associated protein 1A/1B-light chain 3-associated phagocytosis
CSF3R: Colony stimulating factor 3 receptor
UTCαβ: Unconventional CD4^−^CD8^−^ αβ T cells
DCs: Dendritic cells
SOCS2: Suppressor-of-cytokine-2 protein
ICB: Immune checkpoint blockade
PD1: Targeting programmed death 1
PD-L1: Programmed death-ligand 1
CTLA4: Cytotoxic T-lymphocyte-associated protein 4
CTL: Cytotoxic T lymphocytes
Sca-1: Stem cell antigen-1
TAMs: Tumor-associated macrophages
CSF1R: Colony stimulating factor 1 receptor
INs-seq: Intracellular staining and sequencing
Mreg: Regulatory myeloid cells
HLA: Human leukocyte antigen
SGC: Salivary gland carcinoma
CCS: Circular consensus-sequencing
ATAC-seq: Assay for Transposase-Accessible Chromatin using sequencing
TF: Transcriptional factor
3C: Chromatin conformation capture analysis
4C: Circular chromosome conformation capture
5C: Carbon copy chromosome conformation capture
m6A: N6 position
m5C: 5-Methylcytosine
CRISPR: Clustered regularly interspaced short palindromic repeats
